# Anemia and mortality in patients with nondialysis-dependent chronic kidney disease

**DOI:** 10.1186/s12882-018-0925-2

**Published:** 2018-06-11

**Authors:** Heide A. Stirnadel-Farrant, Jiacong Luo, Lata Kler, Borut Cizman, Delyth Jones, Steven M. Brunelli, Alexander R. Cobitz

**Affiliations:** 1 DaVita Clinical Research, Minneapolis, MN USA; 2 GlaxoSmithKline, Collegeville, PA USA; 3 GlaxoSmithKline, Stevenage, Hertfordshire, SG1 2NY UK

**Keywords:** Erythropoietin, Hemoglobin, Iron, Cardiovascular outcomes, Kidney disease

## Abstract

**Background:**

A combination of safety concerns and labeling changes impacted use of erythropoiesis-stimulating agents (ESAs) in renal anemia. Data regarding contemporary utilization in pre-dialysis chronic kidney disease (CKD) are lacking.

**Methods:**

Electronic healthcare records and medical claims data of pre-dialysis CKD patients were aggregated from a large US managed care provider (2011–13). ESA use patterns, characteristics, and outcomes of ESA-treated/untreated patients were quantified.

**Results:**

At baseline, 109/32,308 patients (0.3%) were ESA users. Treated patients were older, had more advanced CKD (58.8% vs 5.4% with stage 4/5 vs 3) and greater prevalence of comorbid diabetes, hypertension, heart failure, and peripheral vascular disease. An additional 266 patients initiated ESA: hemoglobin at initiation was 8–10 g/dL in 193 of these and >10 g/dL in the remainder; 61.7% had stage 4/5 CKD; prevalence of cardiovascular disease was high (50.8% heart failure; 25.2% prior myocardial infarction; 24.1% prior stroke). During follow-up, rates of death and cardiovascular events were higher in baseline ESA users and ESA naives versus non-users.

**Conclusions:**

ESA use in pre-dialysis CKD patients was exceedingly rare and directed disproportionately to older, sicker patients; these patients had high rates of death and cardiovascular events. These data provide context for contemporary use of ESA in pre-dialysis CKD.

**Electronic supplementary material:**

The online version of this article (10.1186/s12882-018-0925-2) contains supplementary material, which is available to authorized users.

## Background

In the years since the publication of the CREATE [1], CHOIR [2], and TREAT [3] clinical studies, there has been a substantial shift in anemia management for patients with chronic kidney disease (CKD). These studies raised concerns regarding erythropoiesis-stimulating agent (ESA) use, including risks of cardiovascular and thrombotic events. The occurrence of these adverse events was associated with ESA dosing practices targeting higher thresholds for hematocrit and hemoglobin (Hb) concentrations. Subsequently, the US Food and Drug Administration-approved labels for ESAs were revised with a Black Box Warning indicating increased risks of cardiovascular events associated with Hb levels greater than 11 g/dL [4]. Several reports have documented rapid changes in treatment patterns of anemia for hemodialysis patients with end-stage renal disease (ESRD) shortly after these events: lower ESA doses, lower Hb levels, greater intravenous iron use [5–9], and increased rates of patients receiving red blood cell transfusions [5, 7].

Although these changes are well documented for ESRD patients, there is a paucity of data describing ESA use patterns among US patients with nondialysis-dependent CKD. Data from two studies reported a decrease in ESA use and Hb concentrations in pre-dialysis CKD from 2005 to 2011 [4, 10] but, to our knowledge, no study has characterized the patterns of treatment in this population after the 2011 ESA label revision. Generalizable data in this regard are important for understanding the current treatment landscape and benchmarking normative clinical practice. With emerging therapies for renal anemia on the horizon, understanding patient characteristics and expected patient outcomes are essential to designing clinical trials. Untreated patients with qualifying degrees of anemia and/or ESA-treated patients are likely to serve as the source population for such trials. This descriptive analysis was conducted to inform in these respects using data from a large, representative, real-world CKD population. Because this was an observational study, it was not possible to assess causality between anemia treatments and outcomes. Therefore, these analyses do not contain formal statistical comparisons.

## Methods

### Data source and patients

We performed a retrospective observational study by examining the database of a large managed care provider that also serves as the insurer of 1.5 million members in the United States. The database contains electronic health records (EHR), medical, and pharmacy claims. EHR data were used to identify patient characteristics, comorbid illnesses, and laboratory values; claims provided visibility to medication utilization and health events during follow-up.

**Fig. 1:**
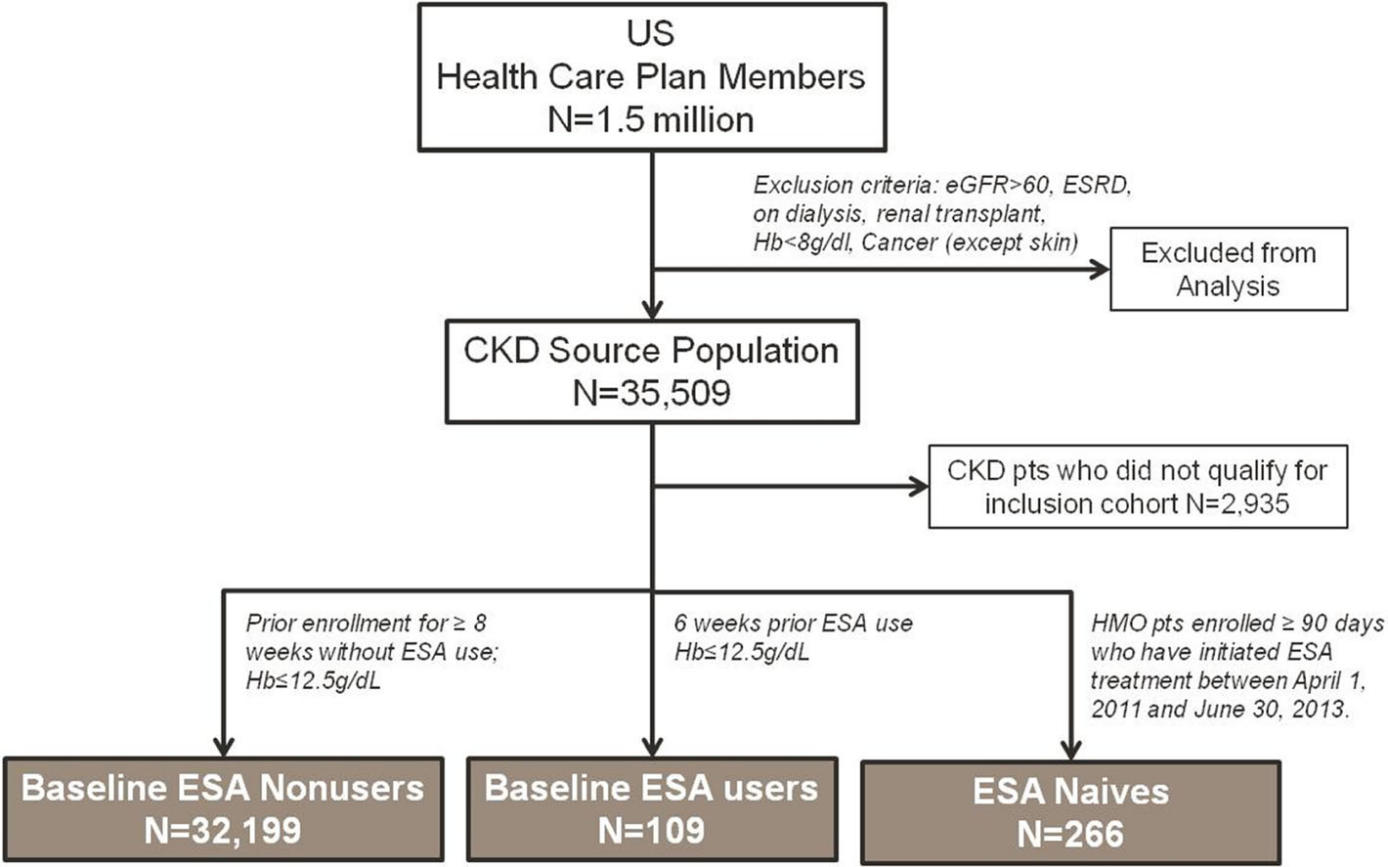
Study CONSORT Diagram. The study patient population was derived from the database of a large health care provider in United States (US). The source population with chronic kidney disease (CKD) was derived by excluding patients who had estimated glomerular filtration rates (eGFR) greater than 60 ml/min/1.73 m^2^, were diagnosed with end-stage renal disease (ESRD), had received renal transplants, had a recent hemoglobin (Hb) measure less than 8 g/dL, or had a diagnosis of cancer (with the exception of skin cancer). The remaining patients were then stratified into study cohorts based on recent Hb measurements and use of erythropoiesis-stimulating agents (ESA)

Patients considered in the study were >18 years old; had eGFR <60 ml/min/1.73m^2^; and had no prior diagnosis of ESRD, treatment with dialysis, or receipt of renal transplant based on claims history. We excluded patients with Hb <8 g/dL on most recent measurement (because anemia of this severity due to CKD alone in patients not on dialysis is unlikely) and those with active malignancy except for common cutaneous cancers (defined as ICD-9 codes 140.x–208.x excluding 172.xx in the prior 2 years). From this source population, we identified three study cohorts based on ESA treatment history (Fig. 1):

- Baseline ESA nonusers: as of index date (01 April 2011), had been enrolled with the provider for ≥8 weeks without use of ESA during this period.
- Baseline ESA users: as of index date (01 April 2011), were enrolled with the provider and had received ESA within the prior 6 weeks, and did not have Hb >12.5 g/dL (to exclude potential influence of patients who were being treated off label).
- ESA naives: between 01 April 2011 and 30 June 2013 had been enrolled in with the provider for ≥90 days without ESA treatment and subsequently received ESA (index date). It is important to note that ESA naives may have been included in other groups at baseline. However, in describing the source population, such patients were not double counted.

We tracked the longitudinal trends of anemia and anemia treatment from index date until death, health plan disenrollment, or study end (31 December 2013). We examined Hb, ESA use, and iron storage indices over time, and prescribed intravenous and oral iron use and dose. For some analyses, we stratified cohorts according to baseline Hb values with thresholds based on ESA product labeling:

- ≤10 versus >10 g/dL for baseline non-users and ESA naives in whom the implied decision is whether to initiate ESA.
- ≤11 versus >11 g/dL among baseline ESA users in whom the implied decision is to continue or discontinue ESA.

We also added a “transitional” category to capture subjects who were just above the Hb target (0.5 g/dL), because most insurance companies do not reimburse for ESA when Hb >10 g/dL. Another category was added to capture subjects who were targeted with higher Hb levels.

### Outcomes

Clinical outcomes considered were cardiovascular events [myocardial infarction (MI), stroke, and hospitalization for congestive heart failure (CHF)] and death. We also considered the major adverse cardiovascular events (MACE) composite outcome, including MI, stroke, and death, and MACE+ which additionally included hospitalization for CHF. Details on outcome definitions and analytic methods are provided in the Additional files.

MI was defined by an inpatient or emergency department claim with a primary ICD-9 code of 410.x. Stroke was defined from inpatient or emergency department claims with primary ICD-9 code 431, 433.x and 434.x. CHF was defined from inpatient claims with primary diagnosis codes 276.6, 276.69, 401.x1 404.x1, 404.x3, 425.xx and 428.xx. Death was determined from health records.

### Analytic methods

Demographics, comorbidities, laboratory values, and treatment patterns of patients were characterized at baseline within Hb strata. For time-varying descriptors, the value measured on or immediately preceding index date was considered. Data are presented as means, standard deviations, medians, interquartile ranges, frequencies and proportions as dictated by data type. During follow-up, ESA and iron doses were considered as monthly averages; laboratory values (Hb, ferritin, and percent saturated transferrin [%TSAT]) were considered as the last value measured in each calendar quarter. All ESA doses were converted and expressed in equivalents of epoetin alfa. Event rates were calculated as the total number of patients who experienced the event over cumulative time at-risk. Exact 95% confidence intervals were determined for Poisson estimated rates. Time-to-event analyses were conducted using Kaplan-Meier methods.

### Ethics and compliance

We used pre-existing, de-identified data. According to 45 CFR part 46 from the United States Department of Health and Human Services this study was exempt from institutional review board approval. We adhered to the Declaration of Helsinki; informed consent was not required. The study was deemed exempt by an Institutional Review Board (Quorum IRB, Seattle, WA).

## Results

### Baseline patient characteristics

Table 1 shows baseline characteristics of the source population and the three cohorts, stratified by Hb values. As of 01 April 2011, there were 35,509 qualifying patients. Of these, 32,199 and 109 qualified for the baseline ESA nonuser and baseline ESA user cohorts, respectively (cross-sectional ESA use prevalence 0.3%). An additional 2935 patients did not qualify for either the baseline ESA user or non-user cohort. Between 01 April 2011 and 30 June 2013, a total of 266 patients met criteria for the ESA naives cohort.

**Table 1:** Baseline characteristics of study cohorts overall and within hemoglobin strata

	Baseline ESA Nonusers				Baseline ESA Users			ESA Naives			Source population
	Hb 8–10 g/dL (*N* = 761)	Hb 10.1–10.5 g/dL (*N* = 780)	Hb >10.5 g/dL (*N* = 30,658)	All (*N* = 32,199)	Hb 8–11 g/dL (*N* = 79)	Hb 11.1–12.5 g/dL (*N* = 30)	All (*N* = 109)	Hb 8–10 g/dL (*N* = 193)	Hb >10 g/dL (*N* = 73)	All (*N* = 266)	All (*N* = 35,509)
Age (years) mean ± SD	78.6 ± 11.5	78.4 ± 11.3	76.1 ± 10.8	76.2 ± 10.8	78.1 ± 12.5	81.2 ± 7.9	78.9 ± 11.5	78.0 ± 10.7	79.1 ± 11.3	78.3 ± 10.9	76.1 ± 10.8
Sex, *n* (%)											
Male	228 (30.0)	220 (28.2)	11,638 (38.0)	12,086 (37.6)	31 (39.2)	9 (30.0)	40 (36.7)	79 (40.9)	23 (31.5)	102 (38.4)	13,456 (37.9)
Female	533 (70.0)	560 (71.8)	19,020 (62.0)	20,113 (62.5)	48 (60.8)	21 (70.0)	69 (63.3)	114 (59.1)	50 (68.5)	164 (61.7)	22,053 (62.1)
Race/ethnicity, *n* (%)											
White	150 (19.7)	188 (24.1)	8821 (28.8)	9159 (28.4)	11 (13.9)	3 (10.0)	14 (12.8)	33 (17.1)	15 (20.6)	48 (18.1)	10,045 (28.3)
Black	58 (7.6)	53 (6.8)	1616 (5.3)	1727 (5.4)	9 (11.4)	0	9 (8.3)	16 (8.3)	6 (8.2)	22 (8.3)	1912 (5.4)
Hispanic	91 (12.0)	69 (8.9)	2108 (6.9)	2268 (7.0)	8 (10.1)	2 (6.7)	10 (9.2)	20 (10.4)	7 (9.6)	27 (10.2)	2425 (6.8)
Asian	26 (3.4)	30 (3.9)	1221 (4.0)	1277 (4.0)	11 (13.9)	1 (3.3)	12 (11.0)	13 (6.7)	5 (6.9)	18 (6.8)	1410 (4.0)
CKD stage, *n* (%)											
3A	322 (42.3)	318 (40.8)	21,869 (71.3)	22,509 (69.9)	8 (10.1)	6 (20.0)	14 (12.8)	19 (9.8)	6 (8.2)	25 (9.4)	24,723 (69.6)
3B	267 (35.1)	310 (39.7)	7389 (24.1)	7966 (24.7)	19 (24.1)	12 (40.0)	31 (28.4)	50 (25.9)	28 (38.4)	78 (29.3)	8829 (24.9)
4	151 (19.8)	141 (18.1)	1343 (4.4)	1635 (5.1)	44 (55.7)	11 (36.7)	55 (50.5)	100 (51.8)	33 (45.2)	133 (50.0)	1849 (5.2)
5	21 (2.8)	11 (1.4)	57 (0.2)	89 (0.3)	8 (10.1)	1 (3.3)	9 (8.3)	24 (12.4)	6 (8.2)	30 (11.3)	108 (0.3)
Diabetes, *n* (%)	449 (59.0)	466 (59.7)	12,850 (41.9)	13,765 (42.8)	55 (69.6)	20 (66.7)	75 (68.8)	128 (66.3)	45 (61.6)	173 (65.0)	15,282 (43.0)
Hypertension, *n* (%)	703 (92.4)	716 (91.8)	26,331 (85.9)	27,750 (86.2)	77 (97.5)	29 (96.7)	106 (97.3)	188 (97.4)	71 (97.3)	259 (97.4)	30,654 (86.3)
CHF, *n* (%)	312 (41.0)	264 (33.9)	6641 (21.7)	7217 (22.4)	40 (50.6)	10 (33.3)	50 (45.9)	97 (50.3)	38 (52.1)	135 (50.8)	7813 (22.0)
MI, *n* (%)	148 (19.5)	141 (18.1)	4428 (14.4)	4717 (14.7)	19 (24.1)	6 (20.0)	25 (22.9)	47 (24.4)	20 (27.4)	67 (25.2)	5182 (14.6)
Stroke, *n* (%)	141 (18.5)	136 (17.4)	4175 (13.6)	4452 (13.8)	14 (17.7)	7 (23.3)	21 (19.3)	49 (25.4)	15 (20.6)	64 (24.1)	4855 (13.7)
PVD, *n* (%)	282 (37.1)	269 (34.5)	7599 (24.8)	8150 (25.3)	33 (41.8)	12 (40.0)	45 (41.3)	92 (47.7)	28 (38.4)	120 (45.1)	8805 (24.8)
Albuminuria, *n* (%)											
<300 mg/dL	273 (35.9)	288 (36.9)	11,084 (36.2)	11,645 (36.2)	26 (32.9)	11 (36.7)	37 (33.9)	56 (29.0)	26 (35.6)	82 (30.8)	12,459 (35.1)
≥300 mg/dL	109 (14.3)	100 (12.8)	1575 (5.1)	1784 (5.5)	23 (29.1)	2 (6.7)	25 (22.9)	69 (35.8)	15 (20.6)	84 (31.6)	1917 (5.4)
Not available	379 (49.8)	392 (50.2)	17,999 (58.7)	18,770 (58.3)	30 (38.0)	17 (56.7)	47 (43.1)	68 (35.2)	32 (43.8)	100 (37.6)	21,133 (59.5)
eGFR, mL/min/1.73m^2^, mean ± SD	40.3 ± 12.6	40.5 ± 11.8	48.3 ± 9.1	47.9 ± 9.4	27.0 ± 11.6	32.8 ± 12.0	28.6 ± 11.9	26.9 ± 11.6	29.5 ± 11.5	27.6 ± 11.6	47.8 ± 9.5
Hb, g/dL, mean ± SD	9.5 ± 0.5	10.3 ± 0.1	13.3 ± 1.4	13.2 ± 1.6	9.9 ± 0.7	11.6 ± 0.4	10.4 ± 1.0	9.2 ± 0.5	11.1 ± 1.4	9.7 ± 1.2	13.1 ± 1.6
Ferritin, ng/mL, p50 [p25, p75]	119 [40, 276]	96 [45, 185]	97 [48, 192]	98 [47, 197]	242 [138, 483]	243 [141, 319]	242 [138, 453]	242 [106, 415]	200 [96, 428]	224 [104, 418]	99 [48, 201]
TSAT, %, mean ± SD	22.7 ± 14.4	22.8 ± 11.5	26.0 ± 10.8	25.5 ± 11.3	28.7 ± 13.5	30.3 ± 15.1	29.0 ± 13.6	25.6 ± 12.2	29.8 ± 14.1	26.6 ± 12.7	25.6 ± 11.4

*Abbreviations*: *CHF* congestive heart failure, *CKD* chronic kidney disease, *CVD* cerebrovascular disease, *eGFR* estimated glomerular filtration rate, *ESA* erythropoiesis-stimulating agent, *Hb* hemoglobin, *MI* myocardial infarction, *PVD* peripheral vascular disease, *SD* standard deviation, *TSAT* transferrin saturation, *unk* unknown

Compared to ESA nonusers, baseline ESA users were on average older, and more likely to have macroalbuminuria, diabetes, hypertension, CHF, coronary artery disease, and peripheral vascular disease (Table 1). Baseline ESA users had higher serum ferritin and TSAT, lower mean eGFR (28.6 vs 47.9 mL/min/1.73m^2^) and, correspondingly, more advanced CKD (50.5% vs 5.1% in stage 4 and 8.3% vs 0.3% in stage 5). Among baseline ESA users, the majority (72.5%) had Hb <11 g/dL; whereas among baseline nonusers, the majority (95.2%) had baseline Hb >10.5 g/dL.

Mean Hb at initiation was 9.7 g/dL for ESA naives. The majority (193; 72.6%) had Hb 8–10 g/dL; the remainder had Hb >10 g/dL. Median ferritin at initiation was 224 ng/ml, mean TSAT was 26.6% and mean eGFR at initiation was 27.6 ml/min/1.73m^2^. Prevalence of diabetes (65%), heart failure (50.8%), prior MI (25.2%), prior stroke (24.1%) and peripheral vascular disease (45.1%) were high at the time of initiation. Severity of CKD (50 and 11.3% had stage 4 and 5 CKD, respectively) and anemia (72.5% had Hb 8–10 g/dL) and prevalence of cardiovascular comorbidities were disproportionate to the source population overall.

### Longitudinal trends of anemia and anemia treatment in CKD patients

**Fig. 2:**
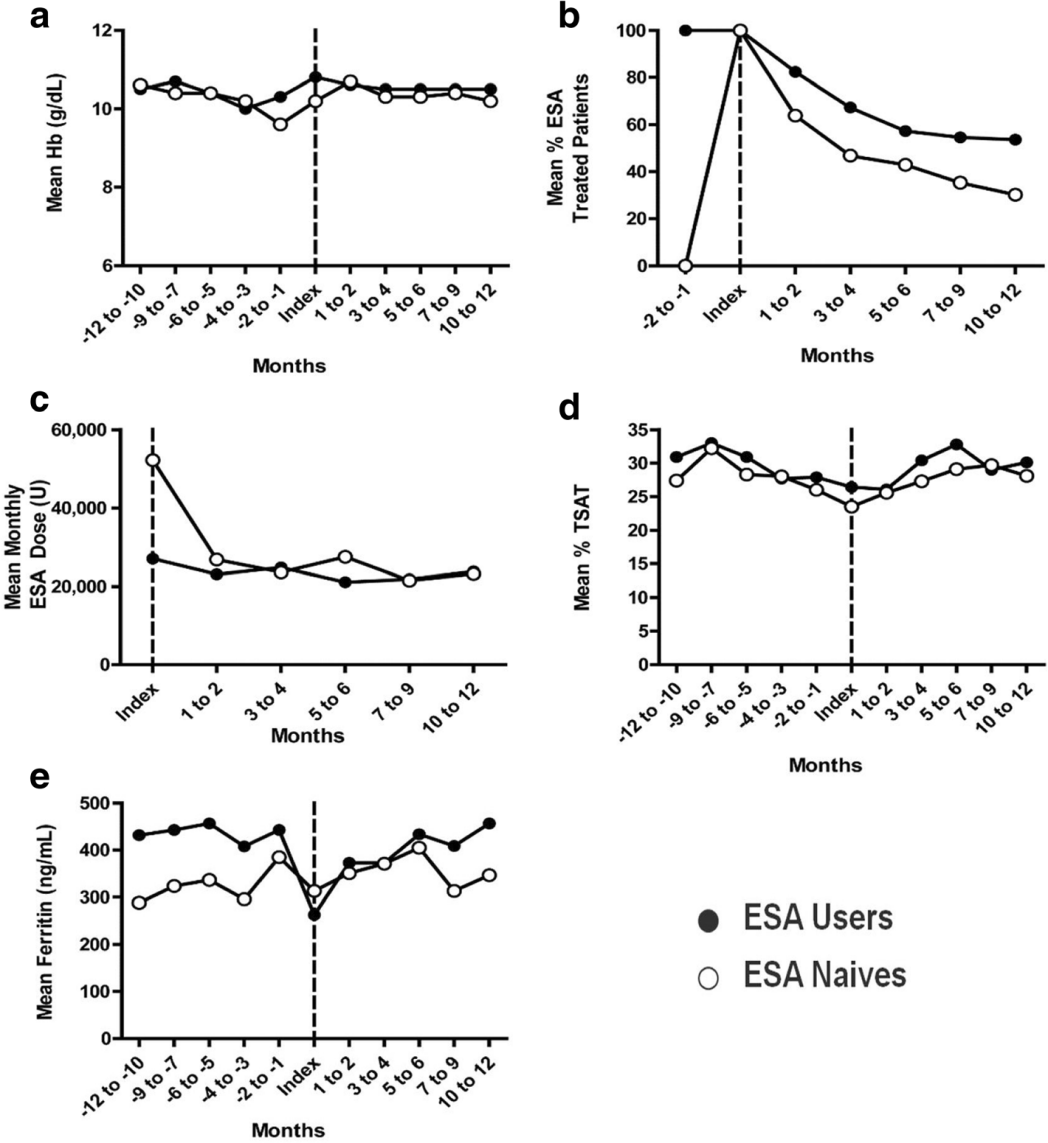
Longitudinal anemia measures and ESA use among baseline ESA users and ESA naives. Mean monthly hemoglobin (Hb) before and after index date (vertical dashed line) are shown in (**a**). Percentage of patients treated with ESA in each cohort is presented in (**b**) and the mean monthly ESA dose among users is shown in (**c**). Percent saturated transferrin (TSAT) (**d**) and serum ferritin (**e**) were measured to assess iron storage

Among baseline ESA users, mean Hb was stable for the 1-year period before and after index date (Fig. 2a and Additional file 1: Table S1). There was gradual discontinuation of ESA such that approximately half of patients discontinued by months 9–10 (Fig. 2b). Mean ESA dose was stable between 20,000 and 30,000 IU per month (Fig. 2c). Iron indices fluctuated over time without a clear trend (Fig. 2d&e).

Among ESA naives, mean Hb gradually decreased in the time leading up to initiation, reaching nadir at 9.6 g/dL in months − 1 to − 2 (Fig. 2a). Hb increased in the peri-initiation period, peaking at 10.7 g/dL 1–2 months post-initiation, followed by a gradual decrease to approximately 10 g/dL over the remainder of follow-up. Among ESA naives, the duration of therapy was short and approximately half of patients discontinued by months 3–4 post-initiation (Fig. 2b). Mean ESA dose at initiation was high (52,163 IU per month) but dropped precipitously, plateauing between 20,000 and 30,000 IU per month 1–2 months later (Fig. 2c). TSAT fell in the year leading up to ESA initiation and rose for the remainder of follow-up. Mean ferritin increased gradually in the 2-year period surrounding ESA initiation, with marked interspersed undulations (Fig. 2d&e).

### Rates of cardiovascular events and death

Among baseline ESA nonusers, rates of MACE, MACE+, death, MI, stroke, and hospitalization for CHF exacerbation were 8.8, 12.0, 3.8, 3.8, 4.2, and 6.9 per 100 patient-years, respectively (Table 2). Among the subset of nonusers with Hb 8–10 g/dL, the corresponding rates were much higher: 21.0, 31.0, 12.0, 7.6, 8.7, and 20.0 per 100 patient years. For all outcomes, there was an Hb concentration-dependent trend of higher rates at lower Hb levels.

**Table 2:** Event rates for outcomes in study cohorts, overall and within hemoglobin strata

	Baseline ESA Nonusers				Baseline ESA Users			ESA Naives		
	Hb 8–10 g/dL	Hb 10.1–10.5 g/dL	Hb >10.5 g/dL	All	Hb 8–11 g/dL	Hb 11.1–12.5 g/dL	All	Hb 8–10 g/dL	Hb >10 g/dL	All
	(*N* = 761)	(*N* = 780)	(*N* = 30,658)	(*N* = 32,199)	(*N* = 79)	(*N* = 30)	(*N* = 109)	(*N* = 193)	(*N* = 73)	(*N* = 266)
eGFR, mL/min/1.73m^2^, mean ± SD	40.3 ± 12.6	40.5 ± 11.8	48.3 ± 9.1	47.9 ± 9.4	27.0 ± 11.6	32.8 ± 12.0	28.6 ± 11.9	26.9 ± 11.6	29.5 ± 11.5	27.6 ± 11.6
MACE										
Event *n*	283	226	5788	6297	28	8	36	55	22	77
Pt-year	1345	1552	68,321	71,218	125	59	183	209	89	298
Mean follow-up, years	1.77	1.99	2.23	2.21	1.58	1.95	1.68	1.08	1.22	1.12
Rate per 100 pt-year (95% CI)	21.04 (18.73, 23.64)	14.56 (12.78, 16.59)	8.47 (8.26, 8.69)	8.84 (8.63, 9.06)	22.45 (15.50, 32.51)	13.66 (6.83, 27.31)	19.64 (14.17, 27.23)	26.32 (20.20, 34.28)	24.67 (16.24, 37.46)	25.82 (20.65, 32.28)
MACE+										
Event *n*	369	301	7515	8185	40	15	55	80	32	112
Pt-year	1203	1428	65,674	68,305	106	48	154	178	78	257
Mean follow-up, years	1.58	1.83	2.14	2.12	1.34	1.61	1.42	0.92	1.07	0.97
Rate per 100 pt-year (95% CI)	30.66 (27.69, 33.95)	21.08 (18.83, 23.60)	11.44 (11.19, 11.70)	11.98 (11.73, 12.25)	37.78 (27.71, 51.51)	30.98 (18.68, 51.38)	35.65 (27.37, 46.43)	44.84 (36.02, 55.83)	40.85 (28.89, 57.77)	43.62 (36.25, 52.50)
Death										
Event *n*	190	136	2573	2899	20	6	26	32	10	42
Pt-year	1533	1734	73,529	76,796	142	61	202	235	102	337
Mean follow-up, years	2.01	2.22	2.40	2.39	1.79	2.03	1.86	1.22	1.40	1.27
Rate per 100 pt-year (95% CI)	12.39 (10.75, 14.28)	7.84 (6.63, 9.28)	3.50 (3.37, 3.64)	3.77 (3.64, 3.91)	14.12 (9.11, 21.88)	9.87 (4.43, 21.97)	12.84 (8.74, 18.86)	13.63 (9.64, 19.27)	9.82 (5.28, 18.24)	12.48 (9.22, 16.88)
MI										
Event *n*	109	107	2587	2803	15	2	17	32	7	39
Pt-year	1441	1629	70,796	73,866	128	59	187	216	96	312
Mean follow-up, years	1.89	2.09	2.31	2.29	1.62	1.96	1.71	1.12	1.31	1.17
Rate per 100 pt-year (95% CI)	7.57 (6.27, 9.13)	6.57 (5.43, 7.94)	3.65 (3.52, 3.80)	3.79 (3.66, 3.94)	11.72 (7.06, 19.44)	3.41 (0.85, 13.63)	9.11 (5.66, 14.65)	14.78 (10.45, 20.90)	7.31 (3.48, 15.33)	12.49 (9.13, 17.09)
Stroke										
Event *n*	123	90	2840	3053	5	2	7	16	11	27
Pt-year	1409	1632	70,395	73,436	138	61	199	224	94	319
Mean follow-up, years	1.85	2.09	2.30	2.28	1.75	2.02	1.82	1.16	1.29	1.20
Rate per 100 pt-year (95% CI)	8.73 (7.32, 10.42)	5.51 (4.49, 6.78)	4.03 (3.89, 4.19)	4.16 (4.01, 4.31)	3.62 (1.51, 8.70)	3.29 (0.82, 13.17)	3.52 (1.68, 7.38)	7.14 (4.37, 11.65)	11.65 (6.45, 21.03)	8.47 (5.81, 12.36)
CHF hospitalization										
Event *n*	261	224	4443	4928	31	14	45	66	23	89
Pt-year	1291	1497	68,827	71,614	112	48	161	188	84	272
Mean follow-up, years	1.70	1.92	2.24	2.22	1.42	1.62	1.47	0.98	1.15	1.02
Rate per 100 pt-year (95% CI)	20.22 (17.91, 22.83)	14.97 (13.13, 17.06)	6.46 (6.27, 6.65)	6.88 (6.69, 7.08)	27.67 (19.46, 39.34)	28.88 (17.11, 48.77)	28.03 (20.93, 37.55)	35.05 (27.53, 44.61)	27.50 (18.27, 41.38)	32.73 (26.59, 40.28)

*Abbreviations*: *CHF* congestive heart failure, *CI* confidence interval, *CKD* chronic kidney disease, *ESA* erythropoiesis-stimulating agent, *Hb* hemoglobin; *hosp* hospitalization, *MI* myocardial infarction, *MACE* major adverse cardiovascular events, *n* event count, *pt* patient, *PVD* peripheral vascular disease, *SD* standard deviation, *yr* year. Event rates 95% confidence intervals are based on exact Poisson estimates. In the case that an exact Poisson regression failed, normal approximation was used. Primary analysis, intent-to-treat

Among baseline ESA users, rates of MACE, MACE+, death, MI, stroke and hospitalization for CHF exacerbation were 20.0, 36.0, 13.0, 9.1, 3.6, and 28.0 per 100 patient-years respectively (Table 2). Rates were higher among patients with lower (8–11 g/dL) versus higher (11.1–12.5 g/dL) baseline Hb, with the exception of CHF for which rates were no different.

Among ESA naives, rates of MACE, MACE+, death, MI, stroke and hospitalization for CHF exacerbation were 26.0, 44.0, 12.0, 12.0, 8.5, and 33.0 per 100 patient-years respectively (Table 2). Among naives with baseline Hb 8–10 g/dL, corresponding rates were 26.0, 45.0, 14.0, 15.0, 7.1, and 35.0 per 100 patient-years. Rates were higher among patients with lower (8–10 g/dL) versus higher (>10 g/dL) baseline Hb, with the exception of stroke, for which rates were higher for patients initiating ESA at higher versus lower Hb (12 vs 7.1 per 100 patient-years). However, differences in event rates by Hb category were less pronounced in ESA naives versus non-users.

Time-to-event curves for MACE and death are presented for all three cohorts in Additional file 2: Figure S1.

We also examined rates of MACE and death in relation to the presence and number of cardiovascular risk factors at baseline (Table 3). Among baseline ESA nonusers, rates of MACE during follow up were incrementally greater for patients with 0, 1, 2, and 3+ risk factors (ranging from 1.8 to 17.0 events per 100 patient-years); a similar pattern was seen for death (ranging 0.36 to 6.9 per 100 patient-years). Among baseline ESA users, there were few patients with zero risk factors. For patients with 1, 2, or 3+ cardiovascular risk factors at baseline, there was a dose-dependent increased risk of MACE (range 2.8 to 33.0 events per 100 patient-years) and death (range 2.8 to 22.0 events per 100 patient-years). ESA naives showed a similar pattern: MACE rates were incrementally greater as the number of risk factors increased from 1 to 2 to 3+ (1.8 to 17.0 events per 100 patient-years) as did rates of death (5.1 to 16 deaths per 100 patient-years).

**Table 3:** Rates for MACE and death based on prevalence of cardiovascular risk factors

Risk Factors	0 factor	Any factor	1 factor	2 factors	≥ 3 factors
Baseline ESA Nonusers					
	(*N* = 2226)	(*N* = 29,973)	(*N* = 10,537)	(*N* = 9334)	(*N* = 10,102)
MACE					
Event *n*	92	6205	1192	1679	3334
Pt-year	5181	66,036	24,811	21,209	20,016
Mean follow-up years	2.33	2.20	2.35	2.27	1.98
Rate per 100 pt-year (95% CI)^a^	1.78 (1.45, 2.18)	9.40 (9.17, 9.63)	4.80 (4.54, 5.08)	7.92 (7.55, 8.30)	16.66 (16.10, 17.23)
Death					
Event *n*	19	2880	511	781	1588
Pt-year	5277	71,519	25,855	22,590	23,073
Mean follow-up years	2.37	2.39	2.45	2.42	2.28
Rate per 100 pt-year (95% CI)^a^	0.36 (0.23, 0.56)	4.03 (3.88, 4.18)	1.98 (1.81, 2.16)	3.46 (3.22, 3.71)	6.88 (6.55, 7.23)
Baseline ESA Users					
	(*N* = 1)	(*N* = 108)	(*N* = 17)	(*N* = 29)	(*N* = 62)
MACE					
Event *n*	0	36	1	6	29
Pt-year	1.1	182	36	58	88
Mean follow-up years	1.05	1.69	2.12	2.00	1.42
Rate per 100 pt-year (95% CI)^a^	NA	19.75 (14.25, 27.38)	2.78 (0.39, 19.70)	10.35 (4.65, 23.03)	32.87 (22.84, 47.30)
Death					
Event *n*	0	26	1	2	23
Pt-year	1.1	201	36	60	106
Mean follow-up years	1.05	1.87	2.12	2.06	1.70
Rate per 100 pt-year (95% CI)^a^	NA	12.91 (8.79, 18.96)	2.78 (0.39, 19.70)	3.34 (0.84, 13.37)	21.78 (14.47, 32.78)
ESA Naives					
	(*N* = 5)	(*N* = 261)	(*N* = 30)	(*N* = 68)	(*N* = 163)
MACE					
Event *n*	0	77	3	19	55
Pt-year	3.9	294	39	84	171
Mean follow-up years	0.78	1.13	1.29	1.24	1.05
Rate per 100 pt-year (95% CI)^a^	NA	26.16 (20.93, 32.71)	7.73 (2.49, 23.97)	22.52 (14.36, 35.30)	32.14 (24.67, 41.86)
Death					
Event *n*	0	42	2	9	31
Pt-year	3.9	333	30	97	197
Mean follow-up years	0.78	1.27	1.30	1.43	1.20
Rate per 100 pt-year (95% CI)^a^	NA	12.62 (9.33, 17.08)	5.11 (1.28, 20.45)	9.28 (4.83, 17.84)	15.76 (11.08, 22.41)

*Abbreviations*: *CI* confidence interval, *eGFR* estimated glomerular filtration rate, *ESA* erythropoiesis-stimulating agent, *Hb* hemoglobin, *MACE* major adverse cardiovascular events, *n* event count, *pt* patient, *yr* year. ^a^Event rates 95% confidence intervals are based on exact Poisson estimates. In the case that an exact Poisson regression failed, normal approximation was used

## Discussion

In this study, we characterized anemia management and outcomes in a contemporary population of pre-dialysis stage 3–5 CKD patients. Among these patients, ESA treatment was rare: 109 out of 35,509 (0.3%) of all patients were being treated with ESA at baseline and only 266 began therapy from 2011 to 2013. There were 761 patients with baseline Hb 8–10 g/dL who were not on ESA therapy, even though treatment would not be inconsistent with product labeling: this is nearly seven times as many patients as were being treated with ESA overall. Among patients with stage 3, 4, and 5 CKD, 0.15, 3.3, and 9.2%, respectively, were receiving ESA at the start of the observation period in this study. Taken in context of previous studies, these results demonstrate that ESA use in pre-dialysis CKD is becoming increasingly conservative. From 2005 to 2009, ESA use dropped from 60 to 46% of all non-dialysis patients with CKD stages 1–5 [4]. ESA treatment rates continued to decline, dropping from 17. 1 to 10.6% and 34.3 to 26.6% in patients with stage 3 and 4 CKD, respectively from 2009 to 2011 [10].

Although it is not possible to rigorously identify individual Hb management goals, it is interesting that of the baseline ESA users, 72.4% had baseline Hb concentrations between 8 and 11 g/dL. Moreover, >75% of ESA naives had Hb concentrations <10 g/dL. After initiation, there was marked discontinuation in ESA use and decrease in mean dose among those remaining on drug such that mean Hb plateaued in the low 10’s g/dL. These data are consistent with the premise that in general, when ESAs were used, therapy was in line with product labeling: initiation for Hb <10 g/dL and titration to Hb no greater than 11 g/dL. Of note, the achieved Hb of just above 10 g/dL is lower than had been reported for patients in 2005–2009 [4].

These data also indicate that ESA treatment in pre-dialysis CKD is directed toward sicker patients. Compared to ESA nonusers, both the baseline ESA users and naives were older with more advanced CKD. Nearly two-thirds of patients initiating ESA therapy (61.3%) did so at stage 4/5. Notably, compared to nonusers, baseline ESA users and naives had greater baseline prevalence of cardiovascular risk factors. The most remarkable was CHF, which was prevalent in 22.4% of ESA nonusers and 45.9 and 50.8% of baseline ESA users and naives, respectively. Rates of prior MI and stroke were also higher among baseline ESA users and naives versus ESA nonusers.

Not surprisingly then, rates of cardiovascular events and mortality during follow-up were substantially higher among the baseline ESA users and ESA naives relative to baseline nonusers for most outcomes. Within each ESA group, outcomes were worse among patients with lower versus higher baseline Hb. These observations may be explained in part by the fact that patients with greater illness tend to be routed toward ESA treatment and also have lower Hb. This hypothesis is supported by our baseline characterization of the patients across cohorts and suggests that severe anemia is a marker of poor prognosis in pre-dialysis CKD. Our analyses demonstrated that the presence of cardiovascular risk factors was incrementally associated with greater MACE and death rates in all patients, a finding that is reflected in both the outcome rates and baseline demographics of the study groups.

The purpose of this study was to present data to set clinical expectations and inform design of randomized trials enrolling patients in the US. With regard to trial design, our population of baseline ESA nonusers—particularly the subset of with baseline Hb 8–10 g/dL— should be a good benchmark of expected event rates in the control group for placebo controlled randomized trials of anemia therapies in pre-dialysis CKD. Likewise, our population of ESA naives should be a good benchmark of expected event rates in the ESA comparator arm of randomized trials of anemia therapies. Our population of baseline ESA users should be representative of expected event rates in the ESA comparator arm of a trial in which patients are randomized to either continue to receive ESA or switch to an alternate anemia therapy. Furthermore, our data can be used to guide expectation about how trial eligibility criteria vis-à-vis cardiovascular risk factors influence expected event rates.

It is worth making explicit that this study was not designed to assess causal effects of ESA use and/or anemia severity on clinical outcomes in the pre-dialysis CKD population. We therefore advise that no causal interpretation be applied to findings. Because our purpose was entirely descriptive, we present event rates and indication of their precision, but purposefully did not conduct formal comparative statistics, nor did we attempt any statistical adjustment. This caveat notwithstanding, there is one observation that bears mention: among ESA naives, rates of all outcomes were higher among patients who initiated at lower versus higher Hb levels with the notable exception of stroke. Rates of stroke were higher among patients who initiated ESA with Hb >10 versus <10 g/dL. This observation is not inconsistent with previously reported findings that aggressive Hb targets for ESA treatment are associated with increased stroke risk [3, 4, 11]. Although we are unable to determine that there is a causal effect of ESA use and higher cardiovascular risk, the utilization of ESA is linked with higher mortality and morbidity. Thus, our findings suggest that there is an increasing trend in current ESA use practice in more careful consideration when and in whom to start ESA (e.g. lower dosage, later introduction of ESAs in non-dialysis CKD population).

There are several limitations to this study. We intentionally excluded patients with cancer as it is likely that anemia management in CKD patients with active malignancies is fundamentally different than among those who are cancer-free [12]; no attempts should be made to generalize our findings to patients with active non-cutaneous malignancies. Although we examined data on iron to the degree possible, IV iron was rarely administered as was prescription oral iron. It may be assumed—particularly considering upward trends in TSAT among ESA naives—that the majority of patients who took iron did so over-the-counter, which precluded our ability to make empiric observations. Finally, our study assessed patients in the United States and care must be taken in extrapolating these findings to other geographies.

## Conclusion

The present analyses demonstrated that from 2011 to 2013, very few patients with pre-dialysis CKD were treated with ESAs for anemia. Those treated with ESAs were older, sicker, and at more advanced stages of CKD. Rates of cardiovascular events and death were greater among ESA-treated patients and those with lower Hb levels.

## Additional files

Additional file 1: **Table S1.** Longitudinal laboratory measurements and ESA doses for baseline ESA users and ESA Naives. (DOCX 52 kb)

Additional file 2: **Figure S1.** MACE and Mortality Rates by ESA use. (TIF 3076 kb)

## Abbreviations

CHF: Congestive heart failure
CKD: Chronic kidney disease
eGFR: estimated glomerular filtration rate
EHR: Electronic health records
ESA: Erythropoiesis-stimulating agents
ESRD: End-stage renal disease
Hb: Hemoglobin
MACE: Major adverse cardiovascular events
MACE+: MACE and includes hospitalization for CHF
MI: Myocardial infarction
TSAT: Transferrin saturation

## References

[1] Drueke TB, Locatelli F, Clyne N, Eckardt KU, Macdougall IC, Tsakiris D, Burger HU, Scherhag A (2006). Normalization of hemoglobin level in patients with chronic kidney disease and anemia. N Engl J Med.

[2] Singh AK, Szczech L, Tang KL, Barnhart H, Sapp S, Wolfson M, Reddan D (2006). Correction of anemia with epoetin alfa in chronic kidney disease. N Engl J Med.

[3] Pfeffer MA, Burdmann EA, Chen CY, Cooper ME, de Zeeuw D, Eckardt KU, Feyzi JM, Ivanovich P, Kewalramani R, Levey AS (2009). A trial of darbepoetin alfa in type 2 diabetes and chronic kidney disease. N Engl J Med.

[4] Regidor D, McClellan WM, Kewalramani R, Sharma A, Bradbury BD (2011). Changes in erythropoiesis-stimulating agent (ESA) dosing and haemoglobin levels in US non-dialysis chronic kidney disease patients between 2005 and 2009. Nephrol Dial Transplant.

[5] Collins AJ, Monda KL, Molony JT, Li S, Gilbertson DT, Bradbury BD (2014). Effect of facility-level hemoglobin concentration on dialysis patient risk of transfusion. Am J Kidney Dis.

[6] Freiburger JK, Ng LJ, Bradbury BD, Kshirsagar AV, Brookhart MA (2012). Changing patterns of anemia management in US hemodialysis patients. Am J Med.

[7] Fuller DS, Pisoni RL, Bieber BA, Gillespie BW, Robinson BM (2013). The DOPPS practice monitor for US dialysis care: trends through December 2011. Am J Kidney Dis.

[8] Pisoni RL, Fuller DS, Bieber BA, Gillespie BW, Robinson BM (2012). The DOPPS practice monitor for US dialysis care: trends through August 2011. Am J Kidney Dis.

[9] Wetmore JB, Peng Y, Monda KL, Kats AM, Kim DH, Bradbury BD, Collins AJ, Gilbertson DT (2015). Trends in Anemia management practices in patients receiving hemodialysis and peritoneal Dialysis: a retrospective cohort analysis. Am J Nephrol.

[10] Thamer M, Zhang Y, Kshirsagar O, Cotter DJ, Kaufman JS (2014). Erythropoiesis-stimulating agent use among non-dialysis-dependent CKD patients before and after the trial to Reduce Cardiovascular Events with Aranesp Therapy (TREAT) using a large US health plan database. Am J Kidney Dis.

[11] Skali H, Parving H-H, Parfrey PS, Burdmann EA, Lewis EF, Ivanovich P, Keithi-Reddy SR, McGill JB, McMurray JJV, Singh AK (2011). Stroke in patients with type 2 diabetes mellitus, chronic kidney disease, and anemia treated with Darbepoetin Alfa: the trial to reduce cardiovascular events with Aranesp therapy (TREAT) experience. Circulation.

[12] Hazzan AD, Shah HH, Hong S, Sakhiya V, Wanchoo R, Fishbane S (2014). Treatment with erythropoiesis-stimulating agents in chronic kidney disease patients with cancer. Kidney Int.

