# Supplementary material for: Anemia and mortality in patients with nondialysis-dependent chronic kidney disease
Source: BMC Nephrol. 2018 Jun 11;19:135. doi: 10.1186/s12882-018-0925-2 (PMC5996482; doi:10.1186/s12882-018-0925-2)
Supplement: Supplementary file 1 — Table S1. Longitudinal laboratory measurements and ESA doses for baseline ESA users and ESA Naives. (DOCX 52 kb) [file 12882_2018_925_MOESM1_ESM.docx]

**Table S1**. Longitudinal laboratory measurements and ESA doses for baseline ESA users and ESA naives

|  | **Month** | | | | | | | | | | |
| --- | --- | --- | --- | --- | --- | --- | --- | --- | --- | --- | --- |
|  | **-12 to -10** | **-9 to -7** | **-6 to -5** | **-4 to -3** | **-2 to -1** | **Index** | **1 to 2** | **3 to 4** | **5 to 6** | **7 to 9** | **10 to 12** |
| **Baseline ESA Users** | | | | | | | | | | | |
| **Hb g/dL**  mean ± SD | 10.5 ± 1.2 | 10.7 ± 1.2 | 10.4 ± 1.2 | 10.0 ± 0.9 | 10.3 ± 1.0 | 10.8 ± 1.3 | 10.6 ± 1.3 | 10.5 ± 1.4 | 10.5 ± 1.4 | 10.5 ± 1.1 | 10.5 ± 1.1 |
| median  [p25, p75] | 10.4  [9.7, 11.2] | 10.7  [9.9, 11.4] | 10.3 [9.7, 11.0] | 10.1  [9.4, 10.5] | 10.3  [9.7, 10.9] | 10.7  10.0, 11.6] | 10.5  [9.7, 11.2] | 10.6  [9.6, 11.3] | 10.5  [9.5, 11.1] | 10.5  [9.5, 11.1] | 10.4  [9.8, 11.3] |
| **ESA Dose ^a^ U/month**  mean ± SD | 17,412 ± 24,742 | 22,518 ± 34,576 | 15,535 ± 23,912 | 15,822 ± 20,312 | 23,768 ± 20,816 | 27,042 ± 28,051 | 23,091 ± 25,900 | 24,794 ± 24,437 | 21,042 ± 22,495 | 21,770 ± 27,879 | 23,765 ± 30,402 |
| median  [p25, p75] | 12,000 [3429, 21,333] | 12,629 [4944,23,571] | 8929  [4286, 14,857] | 11,500 [2286, 20,000] | 23,571 [10,071, 29,286] | 20,000 [10,000, 40,000] | 19,333 [8000, 32,857] | 18,976 [8000, 39,333] | 18,000 [5357, 29,714] | 16,190 [9048, 26,190] | 17,778 [8898, 26,667] |
| **TSAT, %**  mean ± SD | 30.9 ± 16.0 | 33.0 ± 17.1 | 30.9 ± 16.4 | 27.7 ± 12.8 | 27.9 ± 12.2 | 26.4 ± 12.4 | 26.1 ± 15.4 | 30.4 ± 12.2 | 32.8 ± 22.6 | 29.0 ± 11.7 | 30.1 ± 16.2 |
| median  [p25, p75] | 27  [21, 38] | 27  [20, 41] | 26  [20, 39] | 23  [20, 32] | 26  [20, 33] | 22  [16, 33] | 22  [15, 33] | 28  [23, 36] | 26  [19, 44] | 24  [22, 37] | 25  [18, 41] |
| **Ferritin ng/mL**  mean ± SD | 432 ± 536 | 443 ± 481 | 457 ± 544 | 408 ± 423 | 443 ± 453 | 263 ± 261 | 373 ± 354 | 373 ± 432 | 434 ± 457 | 409 ± 447 | 457 ± 374 |
| median  [p25, p75] | 244  [120, 474] | 312  [120, 486] | 288  [109, 576] | 247  [122, 508] | 256  [138, 581] | 163  [86, 315] | 261  [114, 479] | 213  [116, 485] | 259  [119, 732] | 260  [102, 576] | 409  [103, 741] |
| **ESA Naives** | | | | | | | | | | | |
| **Hb g/dL**  mean ± SD | 10.6 ± 1.4 | 10.4 ± 1.1 | 10.4 ± 1.3 | 10.2 ± 1.4 | 9.6 ± 0.9 | 10.2 ± 1.3 | 10.7 ± 1.4 | 10.3 ± 1.3 | 10.3 ± 1.4 | 10.4 ± 1.2 | 10.2 ± 1.2 |
| median  [p25, p75] | 10.3  [9.8, 11.2] | 10.3 [9.7,10.9] | 10.3 [9.5, 11.2] | 10.0  [9.4, 10.9] | 9.4  [8.9, 10.0] | 10.1  [9.2, 11.1] | 10.6  [9.7, 11.5] | 10.4  [9.6, 11.1] | 10.4 [9.3, 11.3] | 10.4  [9.7, 11.2] | 10.1  [9.4, 10.8] |
| **ESA Dose ^a^ U/month**  mean ± SD | 24,424 ± 26,914 | 19,485 ± 28,479 | 27,657 ± 29,803 | 14,955 ± 15,840 | NA | 52,163 ± 45,964 | 26,766 ± 22,635 | 23,550 ± 23,929 | 27,480 ± 25,651 | 21,470 ± 20,682 | 23,151 ± 25,758 |
| median  [p25, p75] | 18,571 [13,333, 30,222] | 13,333 [8000, 18,095] | 20,000 [10,000, 30,857] | 9000  [6345, 19,643] | NA | 40,000 [21,429, 48,000] | 20,000 [14,119, 40,000] | 19,286 [7333, 29,286] | 20,000 [10,000, 38,119] | 16,222 [8237, 28,571] | 16,587 [9524, 28,889] |
| **TSAT, %**  mean ± SD | 27.4 ± 13.4 | 32.2 ± 14.7 | 28.3 ± 13.9 | 28.0 ± 14.5 | 26.0 ± 11.4 | 23.5 ± 10.5 | 25.6 ± 11.8 | 27.3 ± 13.4 | 29.1 ± 15.2 | 29.7 ± 13.8 | 28.1 ± 12.2 |
| median  [p25, p75] | 23  [20, 32] | 29  [23,37] | 25  [20, 34] | 26  [18, 35] | 24  [19, 32] | 23  [15, 30] | 25  [15, 32] | 27  [16, 36] | 25  [19, 35] | 26  [20, 36] | 29  [18, 35] |
| **Ferritin ng/mL**  mean ± SD | 288 ± 277 | 324 ± 320 | 337 ± 348 | 296 ± 331 | 385 ± 436 | 313 ± 375 | 351 ± 372 | 371 ± 474 | 405 ± 464 | 313 ± 322 | 347 ± 319 |
| median  [p25, p75] | 228  [112, 352] | 234 [132,403] | 229  [126, 444] | 211  [85, 350] | 240  [111, 450] | 172  [86, 424] | 239  [68, 467] | 209  [112, 466] | 278  [123, 503] | 213  [92, 4433] | 224  [107, 576] |
| ^a^ Dose calculated for ESA users only.  Abbreviations: ESA, erythropoietin-stimulating agent; Hb, hemoglobin; p25, 25^th^ percentile; p75, 75^th^ percentile; SD, standard deviation; TSAT, saturated transferrin. | | | | | | | | | | | |
