# Supplementary figures and images for: Anemia and mortality in patients with nondialysis-dependent chronic kidney disease
Source: BMC Nephrol. 2018 Jun 11;19:135. doi: 10.1186/s12882-018-0925-2 (PMC5996482; doi:10.1186/s12882-018-0925-2)

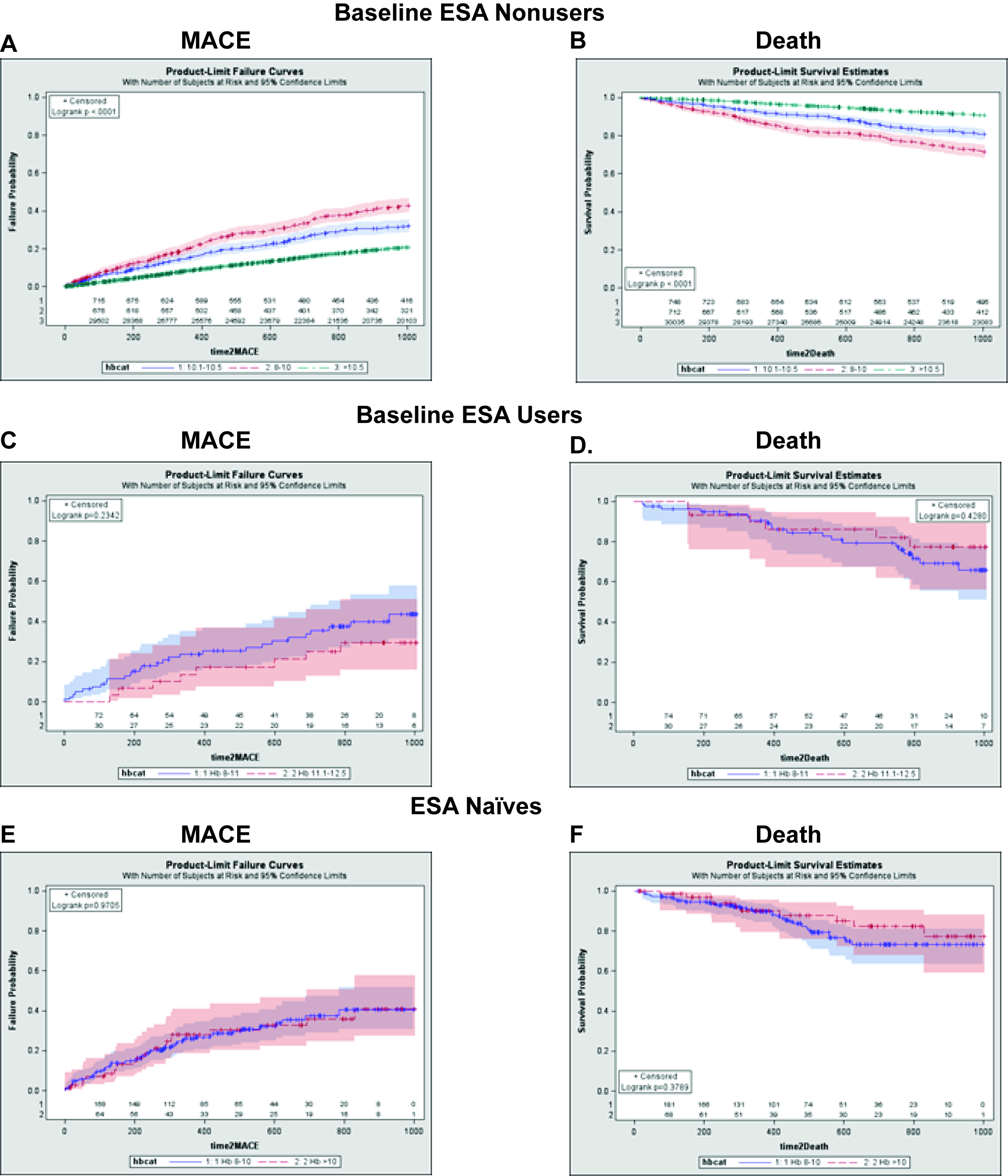

Supplement: Supplementary file 2 — Figure S1. MACE and Mortality Rates by ESA use. (TIF 3076 kb) [file 12882_2018_925_MOESM2_ESM.tif]
